# Predicting overstriding with wearable IMUs during treadmill and overground running

**DOI:** 10.1038/s41598-024-56888-4

**Published:** 2024-03-15

**Authors:** Lauren M. Baker, Ali Yawar, Daniel E. Lieberman, Conor J. Walsh

**Affiliations:** 1 John A. Paulson School of Engineering and Applied Sciences, Harvard University, 150 Western Avenue, Boston, MA 02134 USA; 2 Department of Human Evolutionary Biology, Harvard University, 11 Divinity Avenue, Cambridge, MA 02138 USA

**Keywords:** Musculoskeletal system, Biomedical engineering

## Abstract

Running injuries are prevalent, but their exact mechanisms remain unknown largely due to limited real-world biomechanical analysis. Reducing overstriding, the horizontal distance that the foot lands ahead of the body, may be relevant to reducing injury risk. Here, we leverage the geometric relationship between overstriding and lower extremity sagittal segment angles to demonstrate that wearable inertial measurement units (IMUs) can predict overstriding during treadmill and overground running in the laboratory. Ten recreational runners matched their strides to a metronome to systematically vary overstriding during constant-speed treadmill running and showed similar overstriding variation during comfortable-speed overground running. Linear mixed models were used to analyze repeated measures of overstriding and sagittal segment angles measured with motion capture and IMUs. Sagittal segment angles measured with IMUs explained 95% and 98% of the variance in overstriding during treadmill and overground running, respectively. We also found that sagittal segment angles measured with IMUs correlated with peak braking force and explained 88% and 80% of the variance during treadmill and overground running, respectively. This study highlights the potential for IMUs to provide insights into landing and loading patterns over time in real-world running environments, and motivates future research on feedback to modify form and prevent injury.

## Introduction

Running injuries are prevalent^1^, from muscle strain and joint pain to tendinopathies and bone stress fractures^2,3^. Higher injury rates result from increased training volume and frequency^4^, as the loading thresholds of biological tissues exceed capacity and remodeling/repair rates^5^. Understanding biomechanical risk factors associated with these injuries is an open challenge^1^. While laboratory-based biomechanical evaluations have provided insights regarding potential predictors of running-related injuries, the exact mechanisms of these injuries remain largely unknown due to small sample sizes, limited prospective studies, and limited ability to analyze biomechanical metrics over time outside of a research laboratory. Context-specific gait analysis becomes increasingly important, given that running on a laboratory treadmill does not represent real-world running.

Loading likely contributes to injury development. From cadaver studies^6^, instrumented joint implants^7^, and in vivo bone strain measurements^8^, loading of biological structures (tendon, muscle, ligament, bone) is known to increase microdamage but how to measure this damage remains a challenge. Although the majority of bone loading occurs due to muscle contraction, ground reaction force (GRF) has commonly been used as a surrogate measure of intrinsic loading in the body; however there is yet to be consensus on which GRF metrics, if any, are the most relevant to injury risk^9,10^.

Despite the challenge of understanding how to best quantify loading, there is clear evidence and consensus that running form influences loading^11^. Running re-training to manage and prevent running-related injuries commonly prescribes reducing loading at impact by increasing stride frequency, transitioning from a rear-foot to a fore-foot strike, and reducing overstriding^12^. The term overstriding refers to the horizontal distance that the foot lands in front of the body’s center-of-mass (COM)^13^. While increasing stride frequency has shown to decrease loading metrics^14^, increasing stride frequency has also been associated with increasing metabolic cost^13^. This approach may be more applicable to novice runners with low stride frequencies, but given the coupling to metabolic cost, stride frequency tends to be conserved across speeds for more experienced runners^15^. Speed is therefore manipulated by lengthening strides, which can be achieved by either increasing aerial time or by overstriding. Thus, measuring stride frequency alone may have limitations for understanding loading patterns and its application for providing feedback on better form. Similarly, measuring only foot strike pattern may not provide enough information to understand how a runner is landing and loading. Transitioning from a rear-foot to a fore-foot landing position has been shown to reduce GRF impact magnitude and rate^16^ and has been postulated to reduce injury rate^3^, but conflicting evidence exists^17^. Additionally, fore-foot strikers can still land with a more extended leg position at foot contact, similar to rear-foot strikers, increasing the stiffness of the leg as well as the braking force required to decelerate the body^14^.

Compared to studying the impact of stride frequency and foot strike pattern on GRFs or loading, the effect of overstriding on these metrics remains less studied. Prior work has shown that limb posture at initial contact, which includes measuring overstriding, may influence subsequent loading patterns in stance during running^18^. Further, a previous study varying overstriding during treadmill (TM) running demonstrated a positive correlation between overstriding and braking impulse^13^, a force metric potentially related to injury. Leveraging the geometric relationship between overstriding and lower extremity sagittal segment angles may enable measurement of overstriding outside of the laboratory. Two-dimensional video has been used in outdoor environments to track thigh, shank, and foot angles during overground (OG) running^19^ but this method is limited to the capture volume of the camera and requires time intensive post-processing of images and videos.

Using wearable sensors to measure lower extremity angles and quantify overstriding could help to understand landing patterns and explain loading behavior during real-world running outside of the laboratory. Inertial measurement units (IMUs) are one of the most common wearable sensors for kinematic evaluations, with over 60 publications in the field of running biomechanics alone^20^; however to date, no study has evaluated the ability of IMUs to measure overstriding. Simplifying the usability of wearable sensors in real-world environments is key to understanding the link between biomechanics and running injury risk. Therefore, the objectives of this study were to validate that wearable IMUs alone can predict overstriding during TM and OG running and to demonstrate that such IMU-derived overstriding metrics correlate with braking force metrics^13,21^.

## Methods

### Participants

Ten healthy adult volunteers (5 females; age: 27.9 ± 4.0 yr, height: 1.73 ± 0.9 m, mass: 71.8 ± 15.3 kg, mean ± standard deviation) participated in this study. Runners were recruited from a local 5k race (finish time: 25.5 ± 2.4 min). At the time of study enrollment, participants reported that they currently run for exercise and had no musculoskeletal injuries or disorders. The Harvard Longwood Medical Area Institutional Review Board (IRB) approved the study, all research was performed in accordance with IRB-approved guidelines and regulations, and all participants provided written informed consent.

**Figure 1:**
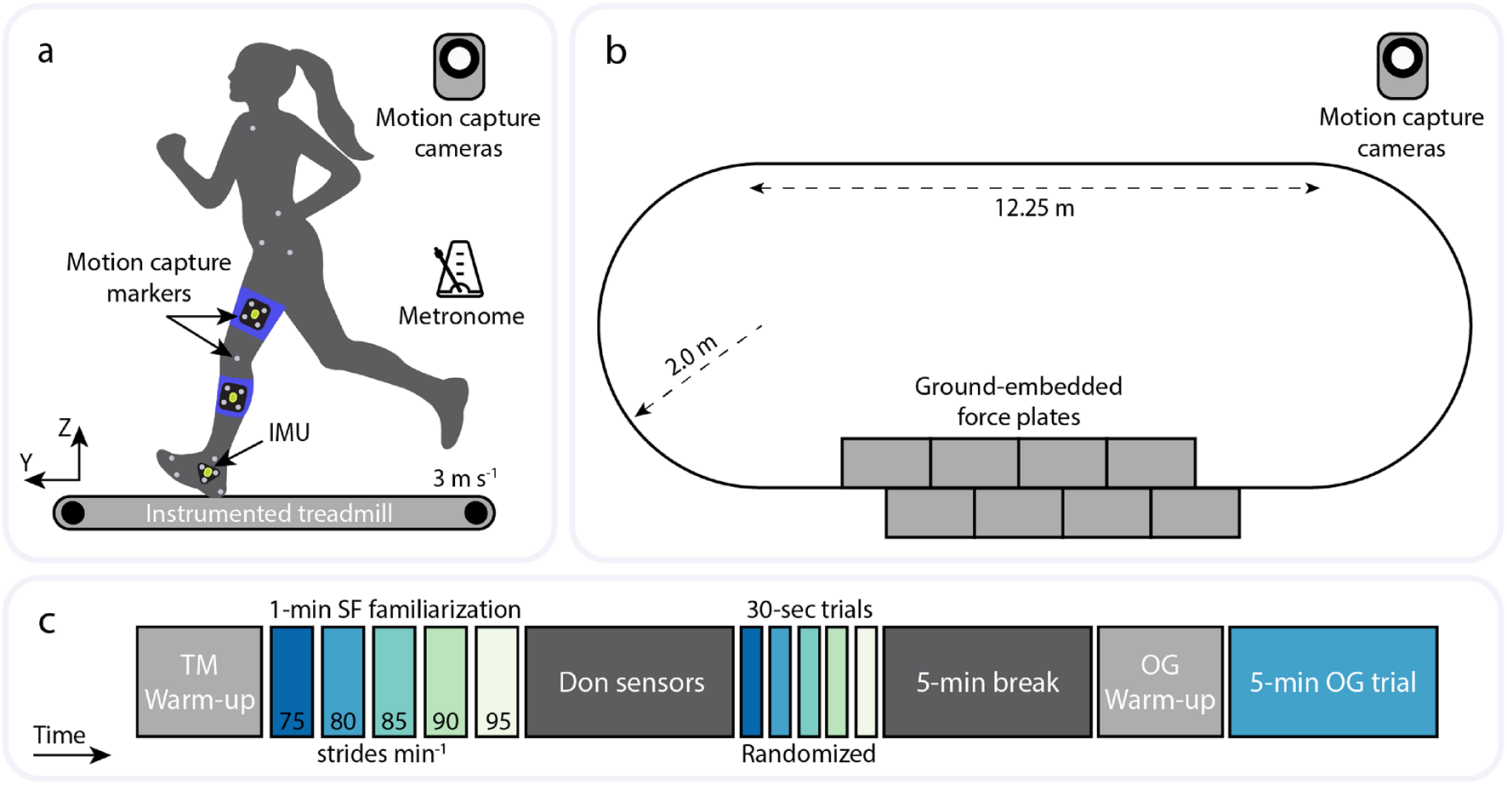
Schematic showing placement of motion capture markers and inertial measurement units (IMUs) worn during running on (**a**) an instrumented treadmill (TM) and (**b**) an overground (OG) laboratory track with ground-embedded force plates. Sagittal segment angles were measured from motion capture and IMUs, and ground reaction forces were collected from force plates. Participants completed TM running at 3 ms^-1^ and matched their strides to a prescribed stride frequency (SF) using a metronome. Participants ran at a self-selected comfortable speed and SF during the OG portion of the protocol. (**c**) Timeline of experimental protocol. Data was recorded during the five 30-s SF-randomized TM trials and one 5-min OG trial.

### Testing protocol

This protocol consisted of a treadmill (TM) running portion and an overground (OG) running portion (Fig. 1). Participants completed 30-s running trials on an instrumented TM set to 3.0 ms^-1^, matching their strides to a metronome set to five different stride frequencies (SF): 75, 80, 85, 90 and 95 strides min^-1^, in randomized order. The last 15 strides were used for analysis. Prior to data collection, participants completed 8 min of warm-up TM running at 3.0 ms^-1^, which included 3 min at their self-selected SF followed by 1 min at each SF to become familiarized with the metronome. Following completion of the TM portion of the protocol, participants took a 5-min break and then completed three to four warm-up laps around a 36-m oval track with embedded force plates on one straightaway. Data was then recorded during a 5-min continuous OG running trial. During this trial, participants were instructed to run at a comfortable self-selected speed and SF. All strides on the force plates within 5% of the median speed over the 5-min OG trial were used for analysis (12.6 ± 1.4 strides).

### Data collection

We collected data from optical motion capture, force plates, and IMUs for all 10 subjects during both TM and OG portions. Reflective markers were placed on anatomical landmarks on the pelvis (left and right iliac crests, left and right anterior superior iliac spines, left and right posterior iliac spines, and left and right greater trochanters), left leg (medial and lateral epicondyles of the femur, medial and lateral malleoli), and left foot (calcaneus, second and fifth metatarsal heads). Marker cluster plates were affixed to the lateral aspects of the left thigh, shank, and foot segments and used as tracking markers. An additional marker was placed on the suprasternal notch to measure running speed during the OG trial. A standing static trial was collected prior to running data collection. Marker data was recorded at 200 Hz using an infrared camera motion capture system (Oqus and Miqus, Qualysis Corp, Gothenburg, Sweden). Three-dimensional GRFs were simultaneously recorded at 2000 Hz using an instrumented treadmill and ground-embedded force plates (Bertec Corp, Columbus, OH). Foot contact times were identified when the vertical GRF exceeded 20 N and were used to determine gait cycle.

Participants wore three wireless IMUs (XSens Dot, Movella Inc, Henderson, NV, weight: 11.2 g, size: 36.3 mm × 30.4 mm × 10.8 mm) on the left thigh, shank, and foot, respectively, which were rigidly attached to the marker cluster plates on these segments. Motion data from the IMUs was recorded at 120 Hz, stored locally on each sensor, and downloaded after each session (accelerometer range: ± 16 g, gyroscope range: ± 2000 ^∘^/s, magnetometer range: ± 8 Gauss). A complementary phone app (XSens Dot, Movella Inc, Henderson, NV) was used to start and stop IMU data recording.

### Data processing

Kinematic and kinetic data from optical motion capture and force plates were analyzed using Visual 3D (C-Motion, Germantown, MD). Motion capture marker data were low-pass filtered using a bidirectional, fourth-order Butterworth filter with a cutoff frequency of 10 Hz. Force data were filtered with a 50 Hz low-pass filter. Besides the manufacturer’s proprietary onboard filtering algorithms, no additional filters were applied to the IMU data.

The 3D orientation data provided by the IMUs was calibrated to a standing pose at the start of the TM trials to better compare to the motion capture data. Since IMU orientation outputs are known to be susceptible to drift, as error is accumulated around the IMU global gravity axis over time, we implemented a different calibration procedure for the OG trial. To manage drift over the 5-min trial, the IMU data was calibrated according to several assumptions^22^. We first assumed that the hip, knee, and ankle were perfect hinge joints, such that all relevant motion of the thigh, shank, and foot occurred in the sagittal plane during running. Based on IMU sensor placement on the lateral aspect of each segment, we assumed that one of the IMU local coordinate axes was aligned with the joint axis and that all relevant segmental rotations were about this axis. At each time frame, we redefined the IMU global reference frame with one axis aligned with the joint axis and another pointed against gravity. We estimated the rotation between the local and the updated global IMU frames, and defined each segment’s sagittal angle as the Euler angle corresponding to rotation about the joint axis.

Across participants, the IMUs started recording after the motion capture data, with a range of delays from 90 to 180 ms. IMU data was synchronized to motion capture data by maximizing the cross-correlation between the segment angles during a calibration maneuver performed before each trial (hard step with the left foot followed by a forward and backward swing of the left leg). Time-aligned IMU and motion capture data were used for all analyses.

### Metric definitions

**Figure 2:**
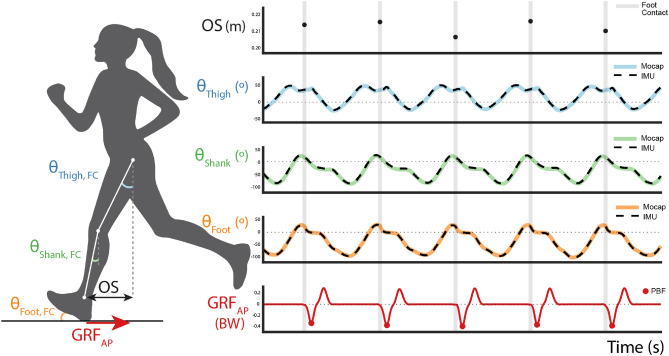
Illustration of data processing and metric definitions from exemplary time-aligned data for a single participant during a treadmill running trial. $\theta _{\text{Thigh}, \text{FC}}$ (green) and $\theta _{\text{Shank}, \text{FC}}$ (blue) represent the sagittal thigh and shank angles relative to vertical at foot contact (FC), where a positive value indicates that the segment is in front of its proximal endpoint. $\theta _{\text{Foot}\ \text{IMU}, \text{FC}}$ (orange) represents the sagittal foot angle relative to the floor at FC, where a positive value indicates a rear-foot strike and a negative value indicates a fore-foot strike. Overstriding (OS) is the horizontal distance between the greater trochanter and the lateral malleolus marker at FC and is geometrically related to $\theta _{\text{Thigh}, \text{FC}}$ and $\theta _{\text{Shank}, \text{FC}}$. Peak braking force (PBF) is computed from the anterior-posterior ground reaction force (GRF_AP_) measured by force plates, shown in red. In the time series plots, shaded gray vertical lines represent FC events. Solid lines represent sagittal segment angles measured from motion capture marker data (Mocap), and black dashed lines represent sagittal segment angles measured from inertial measurement units (IMU). We observed high alignment between the synced Mocap and IMU data.

Several metrics were analyzed in this study (Fig. 2). Overstriding refers to the horizontal distance between the greater trochanter marker and the lateral malleolus marker. To compare across participants, this distance was normalized by leg length. $\theta _{\text{Thigh}, \text{FC}}$, $\theta _{\text{Shank}, \text{FC}}$, and $\theta _{\text{Foot}, \text{FC}}$ refer to the sagittal thigh, shank, and foot angles at foot contact (FC) derived from Visual 3D with respect to the laboratory global frame. Sagittal thigh and shank angles from motion capture are referenced to vertical, with a positive $\theta _{\text{Thigh}, \text{FC}}$ or $\theta _{\text{Shank}, \text{FC}}$ indicating that the segment is in front of its proximal endpoint. Sagittal foot angle from motion capture is referenced to horizontal (e.g. the floor), with positive $\theta _{\text{Foot}, \text{FC}}$ indicating a rear-foot strike and negative $\theta _{\text{Foot}, \text{FC}}$ indicating a fore-foot strike. $\theta _{\text{Thigh}\ \text{IMU}, \text{FC}}$, $\theta _{\text{Shank}\ \text{IMU}, \text{FC}}$, and $\theta _{\text{Foot}\ \text{IMU}, \text{FC}}$ refer to the sagittal thigh, shank, and foot angles at FC derived from IMUs. $\theta _{\text{Thigh}\ \text{IMU}, \text{FC}}$ and $\theta _{\text{Shank}\ \text{IMU}, \text{FC}}$ are measured with respect to vertical, and $\theta _{\text{Foot}\ \text{IMU}, \text{FC}}$ is measured with respect to horizontal. Peak braking force (PBF) refers to the peak of the posterior GRF.

### Statistical analysis

We compared sagittal segment angles computed with IMU orientations to sagittal segment angles from motion capture at foot contact using root mean square error (RMSE). Linear mixed model (LMM) regressions were performed to quantify the relationship between sagittal segment angles at foot contact and overstriding as well as sagittal segment angles at foot contact and PBF during TM and OG running (Table 1).

As overstriding is a global distance measurement, the sum of segment lengths multiplied by the sine of sagittal segment angles would be required to measure overstriding directly. While segment lengths are easily measured using motion capture markers placed at proximal and distal endpoints, this study sought to use IMUs alone to predict overstriding. Therefore we included only sagittal segment angles as predictors in the LMMs.

**Table 1:** Linear mixed model (LMM) regressions for various dependent variables and fixed effects (model predictors) across treadmill (TM) and overground (OG) running conditions.

LMM	Condition	Dependent variable	Fixed effects
1A	TM	Overstriding	$\theta _{\text{Thigh}, \text{FC}}, \theta _{\text{Shank}, \text{FC}}, \theta _{\text{Foot}, \text{FC}}$
2A	OG	Overstriding	$\theta _{\text{Thigh}, \text{FC}}, \theta _{\text{Shank}, \text{FC}}, \theta _{\text{Foot}, \text{FC}}$
3A	TM	PBF	$\theta _{\text{Thigh}, \text{FC}}, \theta _{\text{Shank}, \text{FC}}, \theta _{\text{Foot}, \text{FC}}$
4A	OG	PBF	$\theta _{\text{Thigh}, \text{FC}}, \theta _{\text{Shank}, \text{FC}}, \theta _{\text{Foot}, \text{FC}}$
1B	TM	Overstriding	$\theta _{\text{Thigh}\ \text{IMU}, \text{FC}}, \theta _{\text{Shank}\ \text{IMU}, \text{FC}}, \theta _{\text{Foot}\ \text{IMU}, \text{FC}}$
2B	OG	Overstriding	$\theta _{\text{Thigh}\ \text{IMU}, \text{FC}}, \theta _{\text{Shank}\ \text{IMU}, \text{FC}}, \theta _{\text{Foot}\ \text{IMU}, \text{FC}}$
3B	TM	PBF	$\theta _{\text{Thigh}\ \text{IMU}, \text{FC}}, \theta _{\text{Shank}\ \text{IMU}, \text{FC}}, \theta _{\text{Foot}\ \text{IMU}, \text{FC}}$
4B	OG	PBF	$\theta _{\text{Thigh}\ \text{IMU}, \text{FC}}, \theta _{\text{Shank}\ \text{IMU}, \text{FC}}, \theta _{\text{Foot}\ \text{IMU}, \text{FC}}$

PBF; peak braking force; $\theta _{\text{Thigh}, \text{FC}}$, $\theta _{\text{Shank}, \text{FC}}$, $\theta _{\text{Foot}, \text{FC}}$ ; sagittal thigh, shank, and foot angles at foot contact; IMU; inertial measurement unit.

LMM regressions were chosen to account for repeated measures on the same individuals^13^. These models are known as “mixed” models because they contain both fixed effects and random effects, with the latter accounting for individual differences in response. LMM regressions follow the form: $Y_{i} = X_{i}\beta + Z_{i}\alpha _{i} + \epsilon _{i}$, where $Y_{i}$ is the dependent or response variable, $X_{i}$ is the independent or predictor variable, $\beta$ is the fixed effect coefficient, $Z_{i}$ is the grouping variable, $\alpha _{i}$ is the random effect coefficient, $\epsilon _{i}$ is an error term, and $i = 1,..., N$. Participant identifier was the grouping variable in all models, and *N* was the total number of strides used in the analysis.

All model inputs were standardized as Z scores and fit using the restricted maximum likelihood (REML) estimator in R’s lme4 package. Each LMM was fit as a random-intercept model, so participant-specific intercepts were identified. For the fixed effects, we reported coefficients, 95% confidence intervals (CI), and *p* values. Significance was set at *p* < 0.05. For the random effect, we reported the intraclass correlation coefficient (ICC), which quantifies the proportion of variance explained by the participant identifier. Marginal $R^{2}$ and conditional $R^{2}$ values were calculated to assess model fit. Marginal $R^{2}$ accounts for only the variance of the fixed effects, while conditional $R^{2}$ incorporates variance of both fixed and random effects^23^.

## Results

### Participants varied overstriding during TM and OG running

**Figure 3:**
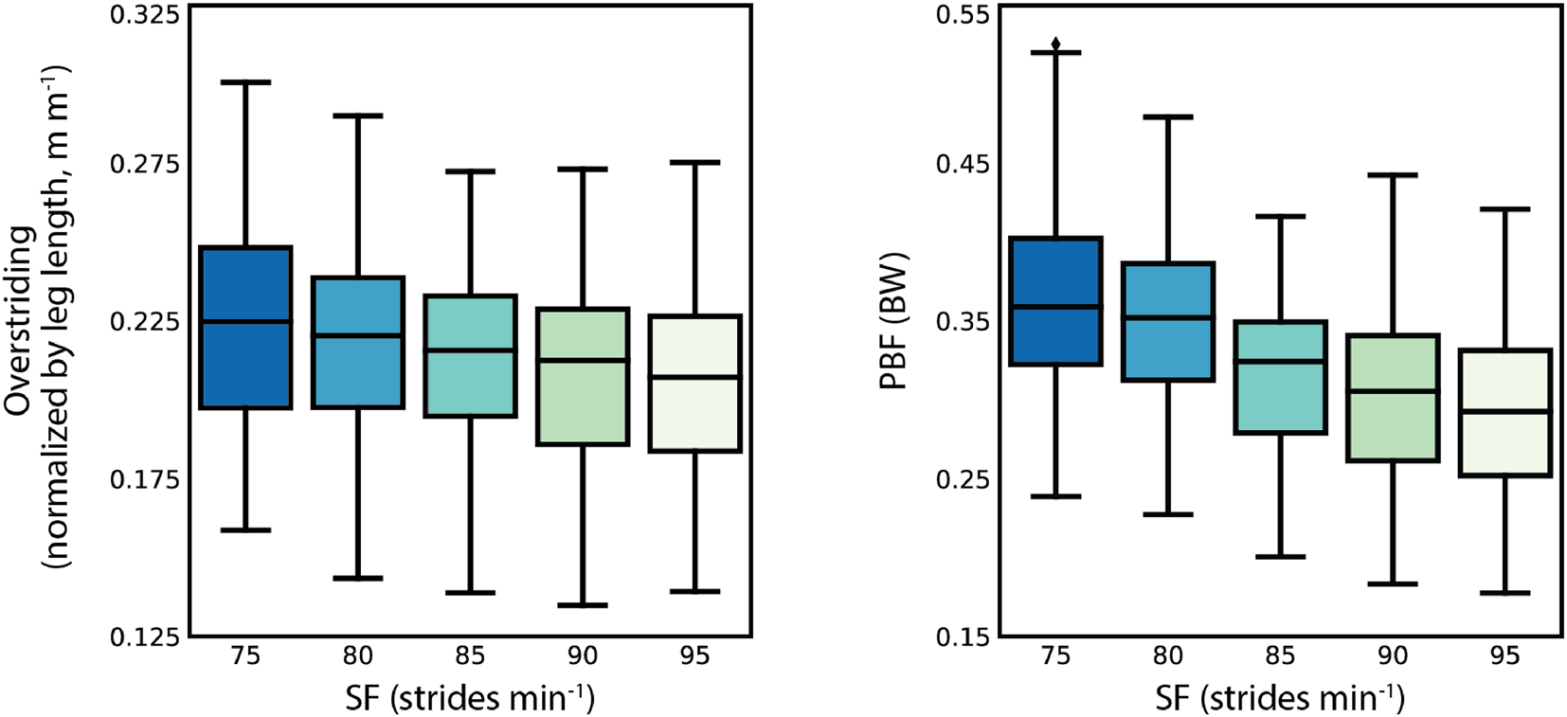
Box and whisker plots showing the distribution of overstriding and peak braking force (PBF) measured at each prescribed stride frequency (SF) during treadmill running. Each box extends from the first to third quartile of the data, with a horizontal line at the median. The whiskers extend from the box to 1.5 times the interquartile range.

During the TM portion of the protocol, participants ran at a constant speed of 3.0 ms^-1^ and were able to match their strides to the prescribed stride frequencies (SF) (Supplementary Table 1). As SF increased, both overstriding and PBF decreased (Fig. 3). The mean range of overstriding for each participant throughout all TM trials was 5.9 ± 1.3 cm (mean ± standard deviation). The mean range of PBF was 0.18 ± 0.05 BW. While the TM trials enabled us to systematically modulate overstriding using a previously published methodology^13^, this work observed stride-to-stride variability in overstriding during OG running at a self-selected comfortable speed (2.9 ± 0.27 ms^-1^) and SF (81 ± 3.5 strides min ^-1^). The mean range of overstriding throughout the OG trial for each participant was 3.8 ± 1.3 cm, and 0.09 ± 0.02 BW for PBF. Overstriding varied less during OG running, which was expected as neither stride frequency, stride length, nor speed was modulated during this portion of the protocol.

We also inspected the ranges of sagittal segment angles at foot contact during TM and OG running. Participants modulated thigh angle by 6.0 ± 1.7^∘^, shank angle by 7.2 ± 1.4^∘^, and foot angle by 8.9 ± 2.1^∘^ during TM running. In general as SF increased, sagittal segment angles at foot contact decreased (Supplementary Table 1). During OG running, participants modulated thigh angle by 3.6 ± 1.6^∘^, shank angle by 4.2 ± 1.4^∘^, and foot angle by 4.7 $\pm$ 2.1^∘^. The range of sagittal segment angles during the OG trial was lower than that of the TM trials. Given the geometric relationship between sagittal segment angles and overstriding, it aligns that the range of both overstriding and sagittal segment angles were lower in OG running than in TM running.

### Sagittal segment angles from motion capture correlated with overstriding during TM and OG running

Thigh and shank angle at foot contact significantly contributed to a model of overstriding during TM running (*p*: <0.001 for both) but not foot angle (*p*: 0.054) (Table 2, LMM 1A). Marginal and conditional $R^{2}$ were 83.0% and 98.8%, respectively (Table 2, LMM 1A). During OG running, thigh and shank angle at foot contact significantly contributed to a model of overstriding (*p*: <0.001 for both) but not foot angle (*p*: 0.202) (Table 2, LMM 2A). Marginal and conditional $R^{2}$ were 86.4% and 99.4%, respectively (Table 2, LMM 2A).

Thigh, shank, and foot angle at foot contact correlated with PBF during TM and OG running. All sagittal segment angles measured from motion capture significantly contributed to a model of PBF during TM running ($\theta _{\text{Thigh}, \text{FC}}$, *p*: <0.001; $\theta _{\text{Shank}, \text{FC}}$, *p*: <0.001; $\theta _{\text{Foot}, \text{FC}}$, *p*: 0.043) (Table 2, LMM 3A) and during OG running ($\theta _{\text{Thigh}, \text{FC}}$, *p*: <0.001; $\theta _{\text{Shank}, \text{FC}}$, *p*: <0.001; $\theta _{\text{Foot}, \text{FC}}$, *p*: 0.018) (Table 2, LMM 4A). For TM running, marginal and conditional $R^{2}$ were 46.2% and 88.3%, respectively (Table 2, LMM 3A). For OG running, marginal and conditional $R^{2}$ were 40.2% and 92.0%, respectively (Table 2, LMM 4A).

### IMUs accurately measured sagittal segment angles during TM and OG running

RMSE between sagittal segment angles measured by motion capture and by IMUs at foot contact during TM running was 2.99^∘^, 4.75^∘^, and 4.33^∘^ for thigh, shank, and foot angles, respectively. During OG running, RMSE was 3.45^∘^, 3.26^∘^, and 4.96^∘^ for thigh, shank, and foot angle, respectively, at foot contact.

### Sagittal segment angles from IMUs correlated with overstriding during TM and OG running

Thigh, shank, and foot angle measured from IMUs at foot contact significantly contributed to a model of overstriding during TM running (*p*: <0.001 for all) (Table 2, LMM 1B). Marginal and conditional $R^{2}$ were 55.7% and 94.7%, respectively (Table 2, LMM 1B). During OG running, thigh and shank angle measured from IMUs at foot contact significantly contributed to overstriding (*p*: <0.001 for both) but not foot angle (*p*: 0.084) (Table 2, LMM 2B). Marginal and conditional $R^{2}$ were 61.8% and 98.2% (Table 2, LMM 2B).

Thigh, shank, and foot angle measured from IMUs at foot contact correlated with PBF during TM and OG running. All sagittal segment angles measured from IMUs significantly contributed to a model of PBF during TM running (*p*: <0.001 for $\theta _{\text{Thigh}, \text{FC}}$, $\theta _{\text{Shank}, \text{FC}}$, and $\theta _{\text{Foot}, \text{FC}}$,) (Table 2, LMM 3B) and during OG running ($\theta _{\text{Thigh}, \text{FC}}$, *p*: <0.001; $\theta _{\text{Shank}, \text{FC}}$, *p*: <0.001; $\theta _{\text{Foot}, \text{FC}}$, *p*: 0.020) (Table 2, LMM 4B). For TM running, marginal and conditional $R^{2}$ were 42.9% and 87.7%, respectively (Table 2, LMM 3B). For OG running, marginal and conditional $R^{2}$ were 35.6% and 79.8%, respectively (Table 2, LMM 4B).

**Table 2:** Linear mixed model (LMM) results.

Dep. Var.	(1)			(2)			(3)			(4)		
	OS TM			OS OG			PBF TM			PBF OG		
Fixed effects	coef.	CI	*p * val.	coef.	CI	*p * val.	coef.	CI	*p * val.	coef.	CI	*p * val.
(A)												
$\theta _{\text{Thigh}, \text{FC}}$	0.61	[ 0.58, 0.63]	<**0.001**	0.53	[ 0.48, 0.58]	<**0.001**	0.68	[ 0.57, 0.80]	<**0.001**	0.70	[ 0.40, 0.63]	<**0.001**
$\theta _{\text{Shank}, \text{FC}}$	0.63	[ 0.61, 0.65]	<**0.001**	0.56	[ 0.52, 0.59]	<**0.001**	0.69	[ 0.60, 0.77]	<**0.001**	0.79	[ 0.50, 0.70]	<**0.001**
$\theta _{\text{Foot}, \text{FC}}$	0.05	[-0.00, 0.11]	0.054	0.08	[-0.04, 0.19]	0.202	-0.25	[-0.50,-0.01]	**0.043**	-0.77	[-1.02,-0.26]	**0.018**
ICC	0.930			0.960			0.830			0.870		
Marginal $R^{2}$	0.830			0.864			0.462			0.402		
Conditional $R^{2}$	0.988			0.994			0.883			0.920		
(B)												
$\theta _{\text{Thigh}\ \text{IMU}, \text{FC}}$	0.52	[ 0.48, 0.57]	<**0.001**	0.29	[ 0.20, 0.37]	<**0.001**	0.69	[ 0.59, 0.79]	<**0.001**	0.35	[ 0.24, 0.46]	<**0.001**
$\theta _{\text{Shank}\ \text{IMU}, \text{FC}}$	0.39	[ 0.37, 0.42]	<**0.001**	0.63	[ 0.52, 0.75]	<**0.001**	0.48	[ 0.42, 0.54]	<**0.001**	0.36	[ 0.26, 0.46]	<**0.001**
$\theta _{\text{Foot}\ \text{IMU}, \text{FC}}$	0.30	[ 0.21, 0.39]	<**0.001**	0.24	[-0.03, 0.52]	0.084	0.48	[ 0.27, 0.69]	<**0.001**	-0.68	[-1.24,-0.11]	**0.020**
ICC	0.880			0.950			0.780			0.690		
Marginal $R^{2}$	0.557			0.618			0.429			0.356		
Conditional $R^{2}$	0.947			0.982			0.877			0.798		

In each LMM, sagittal segment angles at foot contact (FC) are the fixed effects, participant identifier is the random effect, and either overstriding (OS) or peak braking force (PBF) is the dependent variable. Results are shown for data collected during treadmill (TM) and overground (OG) running. The top row (A) reports sagittal segment angles measured from motion capture, denoted as $\theta _{\text{Thigh}, \text{FC}}$, $\theta _{\text{Shank}, \text{FC}}$, and $\theta _{\text{Foot}, \text{FC}}$.. The bottom row (B) reports sagittal segment angles measured by IMUs, denoted as $\theta _{\text{Thigh}\ \text{IMU}, \text{FC}}$, $\theta _{\text{Shank}\ \text{IMU}, \text{FC}}$, and $\theta _{\text{Foot}\ \text{IMU}, \text{FC}}$. *P* values < 0.05 are in bold.

## Discussion

The purpose of this study was to demonstrate that wearable IMUs can predict overstriding during running and then to investigate if such IMU-derived overstriding metrics correlate with braking force metrics. Before using IMU data, we first confirmed that overstriding can be represented by lower extremity sagittal segment angles measured from motion capture, as geometrically expected, during both TM and OG running. Comparing the marginal and conditional $R^{2}$ values of the TM and OG models enables us to understand the predictive performance of the sagittal segment angles for overstriding measurement. We found that $\theta _{\text{Thigh}, \text{FC}}$ and $\theta _{\text{Shank}, \text{FC}}$ correlated with overstriding during TM running, explaining 83% and 99% of the marginal and conditional variance, respectively, and during OG running, explaining 86% and 99% of the marginal and conditional variance, respectively. $\theta _{\text{Foot}, \text{FC}}$ did not significantly contribute to these models (Supplementary Fig. 1). From the marginal variance explained in this dataset, we expect that thigh and shank angles measured from additional participants would show good predictive performance for overstriding.

After demonstrating that sagittal segment angles measured from motion capture are correlated with overstriding, we showed that IMUs can be used to measure these angles. The error between motion capture and IMU measurement for sagittal segment angles at foot contact was less than 5^∘^ for all segments for both TM and OG running, which is similar in magnitude to what has previously been reported^24^. We therefore considered the IMU sagittal segment angles to be reasonable. To demonstrate the potential for assessing overstriding outside of a laboratory environment, we showed that IMUs worn on the thigh and shank can predict overstriding during TM running, explaining 56% and 95% of the marginal and conditional variance, respectively, and during OG running, explaining 62% and 98% of the marginal and conditional variance, respectively. These results are lower than the marginal variance explained by motion capture angles (83% TM and 86% OG). Based on the magnitude of the measurement errors between motion capture and IMU sagittal segment angles during TM and OG running, we expect that the models using IMU angles would have similar but worse performance to those using motion capture.

To further highlight the potential utility of wearable sensors that measure sagittal segment angles, we evaluated the relationship between these angles and braking force, a commonly reported metric in running injury literature. We leveraged the established relationship between overstriding and braking during TM running^13^ to understand how sagittal segment angles first contribute to overstriding and then subsequently to PBF. We found that sagittal segment angles measured from IMUs correlated with PBF during both TM and OG running. $\theta _{\text{Thigh}\ \text{IMU}, \text{FC}}$, $\theta _{\text{Shank}\ \text{IMU}, \text{FC}}$, and $\theta _{\text{Foot}\ \text{IMU}, \text{FC}}$ significantly contributed to a model of PBF, explaining 43% and 88% of the marginal and conditional variance, respectively during TM running and 36% and 80% of the marginal and conditional variance, respectively during OG running. These results are similar to the marginal variance explained by motion capture sagittal segment angles (46% TM and 40% OG). These results are also comparable to recent studies which explained 52% of the variance in cumulative braking impulse during TM running using commercial wearable sensors on the wrist and chest^25^.

While neither $\theta _{\text{Foot}, \text{FC}}$ nor $\theta _{\text{Foot}\ \text{IMU}, \text{FC}}$ significantly contributed to overstriding during OG running, this segment angle did significantly contribute to PBF. The landing angle of the foot may therefore provide additional information to explain braking force, after accounting for overstriding measured by thigh and shank sagittal angles. This finding is similar to a previous study that showed both distance from COM to heel (overstriding) and foot strike angle are kinematic predictors of PBF, explaining 57% of the variance during TM running along with self-selected speed and stride length as predictors^21^. A sub-analysis of our OG data showed that a model with only $\theta _{\text{Thigh}\ \text{IMU}, \text{FC}}$ and $\theta _{\text{Shank}\ \text{IMU}, \text{FC}}$ as predictors explained 43% of the marginal variance of PBF (compared to 36% with the addition of $\theta _{\text{Foot}\ \text{IMU}, \text{FC}}$), while a model with $\theta _{\text{Shank}\ \text{IMU}, \text{FC}}$ as the only predictor still explained 26% of the marginal variance.

Understanding the relative contribution of each segment to overstriding per individual across multiple speeds and slopes would be useful to determine the minimal sensor set required to achieve biomechanically-relevant, robust information on running form. When considering a minimal sensor set, it is helpful to target sensor placement based on which metrics are most desired. For example, using thigh and shank IMUs have been used to predict knee stiffness^26^, a metric found to be related to injury in a prospective study^27^, while also providing information about overstriding and braking force. Runners with tibial stress fractures were found to have higher peak tibial acceleration values compared to uninjured runners^28^, which can be measured with an IMU on the shank^29^, and an additional IMU on the thigh could help elucidate the role of the knee in shock attenuation and injury, especially over a prolonged run^30^.

While this study focused on measuring overstriding with IMUs, recent prospective studies have identified other spatiotemporal and kinematic metrics related to bone stress injuries, namely step rate^31^, vertical excursion^31^, and duty cycle^32,33^ (contact time divided by stride time). Duty cycle has previously been demonstrated to predict PBF, explaining 43% of the variance in a cohort of recreational runners^34^. A sub-analysis of our data showed that duty cycle, $\theta _{\text{Thigh}\ \text{IMU}, \text{FC}}$, and $\theta _{\text{Shank}\ \text{IMU}, \text{FC}}$ significantly contribute to a model of PBF and can explain 59% of the marginal variance during OG running (compared to 43% with only $\theta _{\text{Thigh}\ \text{IMU}, \text{FC}}$ and $\theta _{\text{Shank}\ \text{IMU}, \text{FC}}$ as predictors and 26% with only duty cycle as a predictor). This study ultimately evaluated the relationship between landing position and braking during running, with the assumption that braking is relevant to injury^35^ and therefore landing position (and degree of overstriding) is also relevant to injury. Recent systematic reviews show a lack of consensus on the relationship between braking force and injury^36–38^, but given that bone is susceptible to failure under shear stress^39^, monitoring braking force is likely still relevant to injury^40^. In this study, we measured peak braking force, but other metrics exist, i.e. impulse, time to peak, rate, etc. Developing models to estimate shear loading on the tibia and foot, similarly to previous models for compressive loading^41^, could provide more insight into cumulative loading and bone or tissue damage occurring during running.

In addition to monitoring running form for injury prevention, measuring overstriding and braking using wearable sensors may have applications in running economy^42^ and performance^19^. Deceleration of the COM at impact may influence metabolic cost^42^, indicating that the length of the braking phase may be important to overall running efficiency. However, a study analyzing video-based kinematics and race performance during an elite marathon did not find any differences in overstriding between the final laps^19^. Only two strides per participant could be compared due to limitations in video capture volume of the race course, so using wearable sensors in similar future studies of fatigue and performance could enable a more continuous measurement of overstriding.

Interestingly this same study analyzing race-based kinematics did find differences between genders, with women having greater relative “foot ahead” distance. Although our dataset is not large enough to compare gender groups, we saw that shorter leg lengths resulted in greater overstriding and greater braking, and women tend to have shorter legs compared to the men in this study (0.86 m vs. 0.92 m). This relationship is expected at constant speed TM running but interestingly during OG comfortable speed running, those with shorter legs still exhibited greater PBF. This finding is in agreement with a previous study that found leg length to be a significant contributor to a model of PBF during OG running^43^. More participants would be needed to study gender differences in overstriding and loading patterns.

There were several limitations in this study. This study was conducted in a controlled laboratory environment on level ground to validate IMU-based measurements against an accepted ground truth; however, further study is needed to demonstrate similar results in less controlled spaces, including variable speeds, slopes, and terrains. We found that the IMU errors are not consistent across segments or stride frequencies (Supplementary Table 1), suggesting sensor noise may be influenced by running parameters. Moreover, the sensitivity of these results to IMU placement across longer durations or across days warrants additional study to provide repeatable, reliable measurements of overstriding. Participants were instructed to wear their typical running shoes to eliminate confounding factors from comfort. However, differences in shoe stiffness may introduce errors or inconsistencies in the braking force measurements^44^. In this laboratory evaluation, we used force plates to segment the gait cycle. Detecting foot contact with IMUs has been demonstrated during running^45^ and implementing this gait segmentation would be necessary for outdoor investigations of overstriding.

This study measured only 10 recreational runners, and given that overstriding can occur in both fore-foot and rear-foot strikers, we did not restrict our inclusion to participants with a specific foot strike pattern. During TM and OG running, we found that those who landed with “extreme” angles, i.e. greater than 10° of plantarflexion or greater than 10° of dorsiflexion, exhibited greater overstriding distances than those who landed with a more neutral foot-to-floor angle. A recent study using an unsupervised learning approach to cluster biomechanical data found that collegiate runners with more than 10° of plantarflexion at landing had a higher incidence of bone stress injuries compared to other less-plantarflexed groups^33^. While overstriding was not explicitly reported, we could posit from our results that the excessively plantarflexed group was overstriding more than the groups who landed closer to neutral. More participants representing the continuum of angles would be needed before we can draw conclusions about the impact of foot strike pattern on overstriding and on injury risk^3^. Further, there is currently no consensus on what qualifies as excessive overstriding, so more real-world tracking with wearable sensors and longitudinal studies are needed.

A next step is to use IMUs to predict overstriding in natural running environments. Managing noise and drift in IMU measurements will be necessary for long term use, and practices to improve accuracy have begun to be established^46,47^. Some considerations for measurement accuracy include developing calibration routines to minimize effects of IMU placement or movement due to soft tissue artifacts. In addition to focusing on sensor signal robustness, minimizing the number of sensors needed to provide informative data is important to enable larger scale implementation and data collection, for example within a collegiate cross-country team or community run club. Future work could consider using biofeedback to reduce overstriding and subsequently braking force. Previous work has demonstrated the potential for biofeedback in reducing tibial shock during TM running using visual feedback^48^ and during OG running using music^49^.

While overstriding is correlated with PBF, developing estimates of anterior-posterior GRF for ground-truth comparison in environments outside of the laboratory is important to better understand loading during long distance running. Likely sensor fusion with pressure insoles and individualized machine learning approaches will be needed, in addition to kinematic measures from IMUs^50^. This multi-modal approach has shown promise in estimating vertical^51^ and 3D^52^ GRF metrics and cumulative tibial loading^53^. By establishing which metrics are currently most relevant to injury and which wearable sensors can be used to yield these data, we can begin to conduct more sensorized prospective injury studies and gain more insights into the mechanisms behind running injury development.

### Supplementary Information

Supplementary Information.

## Data Availability

The datasets used and analyzed during this study are available from the corresponding author on reasonable request.

## References

[1] Willwacher S (2022). Running-related biomechanical risk factors for overuse injuries in distance runners: a systematic review considering injury specificity and the potentials for future research. Sports Med..

[2] Lopes AD, Hespanhol LC, Yeung SS, Costa LOP (2012). What are the main running-related musculoskeletal injuries?. Sports Med..

[3] Daoud AI (2012). Foot strike and injury rates in endurance runners. Med. Sci. Sports Exerc..

[4] Malisoux L, Nielsen RO, Urhausen A, Theisen D (2015). A step towards understanding the mechanisms of running-related injuries. J. Sci. Med. Sport.

[5] Hreljac A (2004). Impact and Overuse Injuries in Runners. Med. Sci. Sports Exerc..

[6] Sharkey NA, Hamel AJ (1998). A dynamic cadaver model of the stance phase of gait: Performance characteristics and kinetic validation. Clin. Biomech..

[7] D’Lima DD, Patil S, Steklov N, Slamin JE, Colwell CW (2006). Tibial forces measured in vivo after total knee arthroplasty. J. Arthroplasty.

[8] Lanyon LE, Hampson WG, Goodship AE, Shah JS (1975). Bone deformation recorded in vivo from strain gauges attached to the human tibial shaft. Acta Orthop. Scand..

[9] Schmida EA, Wille CM, Stiffler-Joachim MR, Kliethermes SA, Heiderscheit BC (2022). Vertical loading rate is not associated with running injury, regardless of calculation method. Med. Sci. Sports Exerc..

[10] Milner CE, Foch E, Gonzales JM, Petersen D (2023). Biomechanics associated with tibial stress fracture in runners: A systematic review and meta-analysis. J. Sport Health Sci..

[11] Keast M, Bonacci J, Fox A (2022). Acute effects of gait interventions on tibial loads during running: A systematic review and meta-analysis. Sports Med..

[12] Barton CJ (2016). Running retraining to treat lower limb injuries: A mixed-methods study of current evidence synthesised with expert opinion. Br. J. Sports Med..

[13] Lieberman DE, Warrener AG, Wang J, Castillo ER (2015). Effects of stride frequency and foot position at landing on braking force, hip torque, impact peak force and the metabolic cost of running in humans. J. Exp. Biol..

[14] Heiderscheit BC, Chumanov ES, Michalski MP, Wille CM, Ryan MB (2011). Effects of step rate manipulation on joint mechanics during running. Med. Sci. Sports Exerc..

[15] Cavanagh P, Kram R (1989). Stride length in distance running: Velocity, body dimensions, and added mass effects. Med. Sci. Sports Exerc..

[16] Lieberman DE (2010). Foot strike patterns and collision forces in habitually barefoot versus shod runners. Nature.

[17] Hamill J, Gruber AH (2017). Is changing footstrike pattern beneficial to runners?. J. Sport Health Sci..

[18] Wille CM, Lenhart RL, Wang S, Thelen DG, Heiderscheit BC (2014). Ability of sagittal kinematic variables to estimate ground reaction forces and joint kinetics in running. J. Orthop. Sports Phys. Therapy.

[19] Hanley B, Bissas A, Merlino S (2020). Men’s and women’s world championship marathon performances and changes with fatigue are not explained by kinematic differences between footstrike patterns. Front Sports Active Living.

[20] Mason R (2022). Wearables for running gait analysis: A systematic review. Sports Med..

[21] Napier C, MacLean CL, Maurer J, Taunton JE, Hunt MA (2019). Kinematic correlates of kinetic outcomes associated with running-related injury. J. Appl. Biomech..

[22] Seel T, Raisch J, Schauer T (2014). IMU-based joint angle measurement for gait analysis. Sensors.

[23] Nakagawa S, Schielzeth H (2013). A general and simple method for obtaining R 2 from generalized linear mixed-effects models. Methods Ecol. Evol..

[24] Berner K, Cockcroft J, Morris LD, Louw Q (2020). Concurrent validity and within-session reliability of gait kinematics measured using an inertial motion capture system with repeated calibration. J. Bodyw. Mov. Ther..

[25] Backes A (2020). Predicting cumulative load during running using field-based measures. Scand. J. Med. Sci. Sports.

[26] Stetter BJ, Krafft FC, Ringhof S, Stein T, Sell S (2020). A machine learning and wearable sensor based approach to estimate external knee flexion and adduction moments during various locomotion tasks. Front. Bioeng. Biotechnol..

[27] Messier SP (2018). A 2-year prospective cohort study of overuse running injuries: The runners and injury longitudinal study (TRAILS). Am. J. Sports Med..

[28] Milner CE, Ferber R, Pollard CD, Hamill J, Davis IS (2006). Biomechanical factors associated with tibial stress fracture in female runners. Med. Sci. Sports Exerc..

[29] Reenalda J, Maartens E, Homan L, Buurke JH (2016). Continuous three dimensional analysis of running mechanics during a marathon by means of inertial magnetic measurement units to objectify changes in running mechanics. J. Biomech..

[30] Reenalda J, Maartens E, Buurke JH, Gruber AH (2019). Kinematics and shock attenuation during a prolonged run on the athletic track as measured with inertial magnetic measurement units. Gait Posture.

[31] Kliethermes SA (2021). Lower step rate is associated with a higher risk of bone stress injury: A prospective study of collegiate cross country runners. Br. J. Sports Med..

[32] Malisoux L, Gette P, Delattre N, Urhausen A, Theisen D (2022). Spatiotemporal and ground-reaction force characteristics as risk factors for running-related injury: a secondary analysis of a randomized trial including 800+ recreational runners. Am. J. Sports Med..

[33] Martin JA, Stiffler-Joachim MR, Wille CM, Heiderscheit BC (2022). A hierarchical clustering approach for examining potential risk factors for bone stress injury in runners. J. Biomech..

[34] Bonnaerens S (2021). Relationship between duty factor and external forces in slow recreational runners. BMJ Open Sport Exerc. Med..

[35] Napier C, MacLean CL, Maurer J, Taunton JE, Hunt MA (2018). Kinetic risk factors of running-related injuries in female recreational runners. Scand. J. Med. Sci. Sports.

[36] Ceyssens L, Vanelderen R, Barton C, Malliaras P, Dingenen B (2019). Biomechanical risk factors associated with running-related injuries: A systematic review. Sports Med..

[37] Milner CE, Foch E, Gonzales JM, Petersen D (2022). Biomechanics associated with tibial stress fracture in runners: A systematic review and meta-analysis. J. Sport Health Sci..

[38] Peterson B (2022). Biomechanical and musculoskeletal measurements as risk factors for running-related injury in non-elite runners: A systematic review and meta-analysis of prospective studies. Sports Med. Open.

[39] Turner C, Wang T, Burr D (2001). Shear strength and fatigue properties of human cortical bone determined from pure shear tests. Calcif. Tissue Int..

[40] Gruber A (2023). The impacts cause injury hypothesis: Running in circles or making new strides?. J. Biomech..

[41] Matijevich ES, Branscombe LM, Scott LR, Zelik KE (2019). Ground reaction force metrics are not strongly correlated with tibial bone load when running across speeds and slopes: Implications for science, sport and wearable tech. PLoS ONE.

[42] Moore IS (2016). Is there an economical running technique? A review of modifiable biomechanical factors affecting running economy. Sports Med..

[43] Garcia MC (2023). Preferred temporal-spatial parameters, physical maturation, and sex are related to vertical and braking forces in adolescent long-distance runners. Sports Biomech..

[44] Logan S, Hunter I, Hopkins JT, Feland JB, Parcell AC (2010). Ground reaction force differences between running shoes, racing flats, and distance spikes in runners. J. Sports Sci. Med..

[45] Mo S, Chow DH (2018). Accuracy of three methods in gait event detection during overground running. Gait Posture.

[46] Al Borno M (2022). OpenSense: An open-source toolbox for inertial-measurement-unit-based measurement of lower extremity kinematics over long durations. J. Neuroeng. Rehabil..

[47] Falbriard M, Meyer F, Mariani B, Millet GP, Aminian K (2020). Drift-free foot orientation estimation in running using wearable IMU. Front. Bioeng. Biotechnol..

[48] Crowell HP, Milnert CE, Hamill J, Davis IS (2010). Reducing impact loading during running with the use of real-time visual feedback. J. Orthop. Sports Phys. Ther..

[49] Van den Berghe P, Lorenzoni V, Derie R, Six J, Gerlo J, Leman M, De Clercq D (2021). Music-based biofeedback to reduce tibial shock in over-ground running: A proof-of-concept study. Sci. Rep..

[50] Edwards WB (2018). Modeling overuse injuries in sport as a mechanical fatigue phenomenon. Exerc. Sport Sci. Rev..

[51] Wouda FJ (2018). Estimation of vertical ground reaction forces and sagittal knee kinematics during running using three inertial sensors. Front. Physiol..

[52] Scheltinga BL, Kok JN, Buurke JH, Reenalda J (2023). Estimating 3D ground reaction forces in running using three inertial measurement units. Front. Sports Active Living.

[53] Elstub L (2022). Tibial bone forces can be monitored using shoe-worn wearable sensors during running. J. Sports Sci..

