# Supplementary material for: Predicting overstriding with wearable IMUs during treadmill and overground running
Source: Sci Rep. 2024 Mar 15;14:6347. doi: 10.1038/s41598-024-56888-4 (PMC10942980; doi:10.1038/s41598-024-56888-4)
Supplement: Supplementary file 1 — Supplementary Information. [file 41598_2024_56888_MOESM1_ESM.pdf]

Predicting overstriding with wearable IMUs during treadmill and overground running

Lauren M. Baker[1], **Ali Yawar**[2], **Daniel E. Lieberman**[2], **and Conor J. Walsh**[1,*]

[1]John A. Paulson School of Engineering and Applied Sciences, Harvard University, 150 Western Avenue, Boston, MA 02134, USA
[2]Department of Human Evolutionary Biology, Harvard University, 11 Divinity Avenue, Cambridge, MA 02138, USA
[*]walsh@seas.harvard.edu

SUPPLEMENTARY MATERIAL

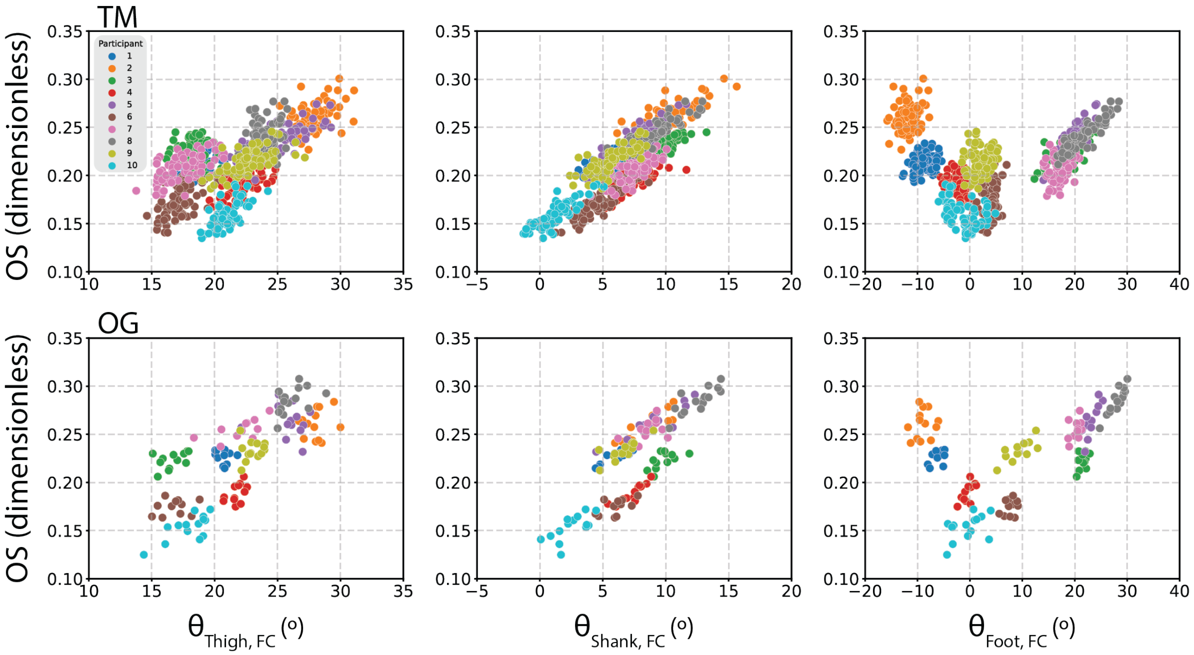

Supplementary Figure 1. Scatterplot of overstriding (OS) vs. sagittal segment angles at foot contact ($\theta_{\text{Thigh, FC}}$, $\theta_{\text{Shank, FC}}$, $\theta_{\text{Foot, FC}}$) for all 10 participants during treadmill (TM) running (top row) and overground (OG) running (bottom row). All data presented are from motion capture. OS is normalized by leg length and thus dimensionless.

Prescribed SF $[strides\ min^{-1}]$	TM 75	TM 80	TM 85	TM 90	TM 95	OG SS
Actual SF	75.4 ± 0.48	80.2 ± 0.30	84.9 ± 0.21	90.0 ± 0.17	94.7 ± 0.33	81.0 ± 3.35
Overstriding	0.22 ± 0.03	0.22 ± 0.03	0.21 ± 0.03	0.21 ± 0.03	0.20 ± 0.03	0.23 ± 0.04
PBF $[BW]$	0.36 ± 0.06	0.35 ± 0.05	0.32 ± 0.05	0.30 ± 0.05	0.29 ± 0.05	0.28 ± 0.04
$\theta_{\text{Thigh, FC}}$ [°]	22.2 ± 3.97	21.6 ± 3.71	21.2 ± 3.50	21.1 ± 3.41	21.0 ± 3.24	22.1 ± 3.98
$\theta_{\text{Thigh IMU, FC}}$ [°]	25.1 ± 4.52	23.4 ± 4.45	23.0 ± 4.17	22.5 ± 3.79	23.2 ± 3.68	24.1 ± 4.32
$\theta_{\text{Shank, FC}}$ [°]	7.85 ± 2.91	7.30 ± 2.69	6.81 ± 2.73	6.17 ± 2.73	5.67 ± 2.79	7.79 ± 2.99
$\theta_{\text{Shank IMU, FC}}$ [°]	11.9 ± 2.50	11.6 ± 2.71	10.3 ± 3.39	10.6 ± 2.66	9.80 ± 2.76	7.72 ± 4.87
$\theta_{\text{Foot, FC}}$ [°]	6.24 ± 13.7	6.36 ± 12.3	6.51 ± 11.4	5.89 ± 11.1	5.16 ± 10.9	9.69 ± 12.8
$\theta_{\text{Foot IMU, FC}}$ [°]	6.20 ± 16.9	4.85 ± 15.7	4.53 ± 15.1	3.18 ± 14.2	2.40 ± 13.2	9.72 ± 13.4

Supplementary Table 1. Mean ± s.d. kinematic and kinetic variables during treadmill (TM) running at prescribed stride frequencies (SF) and overground (OG) running at self-selected (SS) SF for all 10 participants. Overstriding distance is normalized by leg length and thus dimensionless. Reported sagittal segment angles are measured at foot contact (FC) by motion capture markers and inertial measurement units (IMU).
